# Relationship of influenza virus infection to associated infections in children who present with influenza-like symptoms

**DOI:** 10.1186/s12879-016-1642-8

**Published:** 2016-06-18

**Authors:** Yitzchok M. Norowitz, Stephan Kohlhoff, Tamar A. Smith-Norowitz

**Affiliations:** Department of Pediatrics, Division of Infectious Diseases, State University of New York Downstate Medical Center, Brooklyn, NY 11203 USA

**Keywords:** Influenza virus, Influenza virus vaccine, Primary care setting

## Abstract

**Background:**

Influenza virus is a major health care burden and is associated with significant morbidity and mortality. Data on morbidity and complications (pneumonia, otitis media) related to influenza virus infection in primary care settings are limited with reports mainly obtained from hospital settings. We assessed the prevalence of complications from viral/bacterial infections in influenza- positive compared with influenza- negative children presenting with influenza-like illness (ILI) in a primary care setting.

**Methods:**

This retrospective, practice-based chart review studied complications from viral/bacterial infections in 255 children and adolescents (females/males, 1-21 years) who presented with ILI. We also compared the prevalence of complications by influenza vaccination status between influenza positive (*N* = 32/121) and influenza negative (*N* = 50/134) cases (2013-2015). Comparisons for categorical variables were made using chi-squared tests.

**Results:**

The prevalence of complications was similar in influenza positive (18/121) and influenza negative (22/134) patients (*P* = NS). Patients presenting with ILI, who were vaccinated, were less likely to test positive for influenza compared with patients who were not vaccinated (*P* = 0.064). However, prevalence of infections was similar in both groups based on vaccination status. We did not find any effect of type of health insurance on influenza status (*P* > 0.05)

**Conclusion:**

Common respiratory complications of seasonal influenza did not differ in influenza positive compared with influenza negative patients. Vaccination with influenza vaccine may result in decreased duration or severity of symptoms, and remains an important public health intervention. In primary care settings, determination of influenza status may be an important tool for clinicians to predict the likelihood of complications.

## Background

Influenza virus is an infectious human respiratory pathogen [1] that causes seasonal infections [1], and is responsible for approximately 3-5 million clinical infections and 250,000-500,000 fatal cases annually [2]; it is largely spread as an aerosol [1]. It is characterized by the sudden onset of high fever, cough, headache, malaise, and inflammation of the upper respiratory tract [1]; symptoms and fever may persist for 7 to 10 days [1]. Even though people of all ages are affected, the prevalence is highest in school-age children [1]; severity is greatest in infants and the elderly [1]. Influenza A and B viruses are the most common causes of influenza-like illness (ILI) [1], but other pathogens (Influenza C, Parainfluenza virus) also cause ILI [1].

Influenza virus is a significant health care burden and is associated with morbidity and mortality [1]. It is well established that Influenza can temporarily suppress host immune defenses, leading to bacterial complications [1]. Innate and adaptive immune responses are activated during Influenza infection and contribute to infection and viral clearance [3].

Prior literature has investigated the course of seasonal influenza infection among pediatric populations, which are reported mainly from hospital settings or emergency rooms [4]. Heikkinen, *et al* reported a prospective study designed to determine the total burden of influenza in children in the community [5]. However, there are few observations regarding the course of seasonal influenza infection or its complications and co-morbidities from viral/bacterial infections in primary care settings [4]. The purpose of this retrospective chart review was to access the prevalence of complications from viral or bacterial infections in influenza-positive compared with influenza-negative children presenting with ILI in a primary care setting. In addition, we compared the prevalence of complications by influenza vaccination status between the two groups. Determination of influenza status may be an important tool for clinicians to predict the likelihood of complications in the primary care setting.

## Methods

### Setting and study population

All data for this retrospective cohort study were obtained from an electronic medical record data base of a private outpatient pediatric practice in Brooklyn, New York; the study period ran between September 2013 and April 2015. The SUNY Downstate Medical Center Institutional Review Board approved this study without the need for written informed consent because the data lacked patient identifiers.

### Assay: Rapid Influenza A & B diagnostic test

The study population consisted of patients presenting with symptoms of influenza (*N* = 255). Inclusion criteria for performing a rapid influenza diagnostic test (OSOM Influenza A & B Test; Sekisui Diagnostics, LLC, San Diego, CA) had to meet the CDC definition of uncomplicated influenza illness signs and symptoms, including fever, myalgia, headache, malaise, nonproductive cough, sore throat, and rhinitis [6]. Cases were defined as an individual presenting in primary care with an acute respiratory illness and tested positive for Influenza A or B or both A and B. Control children were defined as individuals presenting with symptoms in the same period that were swabbed and tested negative for influenza. The specimens (nasopharyngeal swab) were processed at the pediatrician’s office according to manufacturer’s recommendations. Positive and negative test results were determined by looking for pink to purple lines in the test line region, indicating an A, B, or A and B positive result.

### Confounding variables and complications

We selected factors previously associated with possible symptoms associated with Influenza Virus infection [6]. Variables measured included insurance status and co-payment amount, date of birth, date of age at exam, gender, BMI, BMI percentile, asthma status, influenza status, fever, fever duration, month of illness, anti-viral treatment, Influenza Virus vaccination (FluZone Quadrivalent Influenza vaccine (Sanofi Pasteur, Paris, France) or FluMist Quadrivalent Influenza vaccine live, intranasal (MedImmune, Astra Zeneca, London, U.K.)), presence of pneumonia, conjunctivitis, enteritis, Group A streptococcal (GAS) tonsillitis, nasopharyngitis, and/or otitis media (+/- ear drum rupture). Complications (pneumonia, otitis media, conjunctivitis) were recorded within 30 days following evaluation for ILI.

### Demographics: Population characteristics

Two hundred and fifty five patients were selected for this study who presented with ILI. The mean age (yrs) at the time of office visit was 9.7 ± 7.6; 51 % of patients were males and 49 % were females. Patients >21 years of age were excluded. 121 patients tested positive for influenza, while 134 control patients tested negative for influenza. The number of vaccinated patients who tested influenza positive (*N* = 32/121) was lower than the number of vaccinated patients who tested influenza negative (*N* = 50/134). The number of unvaccinated patients who tested influenza positive (*N* = 89/121) was slightly higher than the number of unvaccinated patients who tested influenza negative (*N* = 84/134). The adjusted vaccine effectiveness (VE) estimate for the influenza seasons 2013-2014 and 2014-2015 were 51 % and 23 %, respectively [7].

### Epidemiology of influenza infections in the study population

**Fig. 1:**
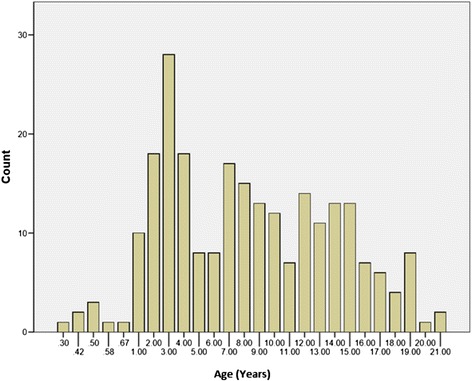
Epidemiology of influenza infections in the study population: number of ILI visits according to age in years. Bars depict the absolute frequency of ILI-visits. Most ILI visits were reported for age 3

**Fig. 2:**
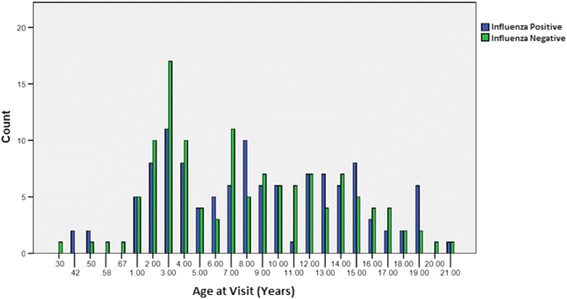
Epidemiology of influenza infection in the study population: number of ILI visits according to age and influenza status. Bars depict the absolute frequency of ILI-visits. Most influenza positive patients were reported for age 3. Blue bar: influenza positive. Green bar: influenza negative

**Fig. 3:**
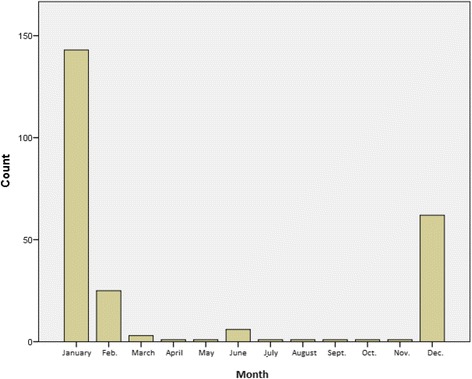
Epidemiology of influenza infections in the study population: number of ILI visits by month of the year. ILI visits increased between the months of December and February, and peaked in the month of January

Number of ILI visits by age is shown in Fig. 1. Most ILI visits were reported for age 3. The number of ILI visits by age and influenza status is shown in Fig. 2. Most influenza positive patients were reported for age 3. The number of ILI visits by month of year is shown in Fig. 3. ILI visits increased between the months of December and February, and peaked in the month of January.

### Type of insurance and co-payment amount

We evaluated the effect of the type of insurance and co-payment amounts on the likelihood of seeking medical treatment with influenza infection. Patient insurance type included either HMO, PPO, government funded (managed care), medicaid or cash. 53 % who tested influenza positive were required to pay a co-payment ($10-$50) for their doctor’s visit, while 47 % of patients who tested influenza positive were not required to pay a co-payment ($0) for their doctor’s visit (*P* = 0.194) (Table 1).

**Table 1:** Insurance plan information for patients presenting with ILI

Type of insurance	Frequency
HMO	177
PPO	4
Government funded (Managed Care)	252
Medicaid (Straight)	6
Cash (self-pay)	26

Insurance type did not significantly affect influenza status (*P* = 0.194)

HMO: Health maintenance organization

PPO: Preferred provider organization

### Statistical analysis

The baseline characteristics of patients with and without Influenza were examined. Comparisons for categorical variables were done using chi square tests. All data and statistical analysis were performed using IBM SPSS software (IBM SPSS Statistics for Windows, Version 22.0, Armonk, NY). A two-sided P-value <0.05 was considered significant. Data are presented as medians, SDs, interquartile ranges (IQRs), or ranges.

## Results

### Prevalence of disease manifestations and complications in influenza positive patients

Patients who tested positive for either Influenza A and B were similar to control patients with respect to pneumonia (*P* = 0.736), nasopharyngitis (*P* = 0.060), Otitis Media (*P* = 0.825), Group A Streptococcal Tonsillitis (*P* = 0.384), Conjunctivitis (*P* = 0.805), and Enteritis (*P* = 0.452) (Table 2).

Patients who tested positive for Influenza A were similar to control patients with respect to pneumonia (*P* = 0.914), nasopharyngitis (*P* = 0.143), Otitis Media (*P* = 0.762), Group A Streptococcal Tonsillitis (*P* = 0.502), Conjunctivitis (*P* = 0.978), and Enteritis (*P* = 0.681) (Table 2).

Patients who tested positive for Influenza B were similar to control patients with respect to pneumonia (*P* = 0. 534), nasopharyngitis (*P* = 0.468), Otitis Media (*P* = 0.738), Group A Streptococcal Tonsillitis (*P* = 0.396), Conjunctivitis (*P* = 0.460), and Enteritis (*P* = 0.350) (Table 2).

**Table 2:** Prevalence of disease manifestations and complications in influenza positive patients

	Pneumonia	Nasopharyngitis	Otitis Media	Group A Streptococcus Tonsillitis	Conjunctivitis	Enteritis
Influenza A + B	0.016	0.297	0.132	0.099	0.024	0.033
Influenza A	0.018	0.305	0.129	0.101	0.027	0.037
Influenza B	0.00	0.277	0.111	0.055	0.000	0.000
Influenza negative	0.022	0.410	0.141	0.134	0.029	0.052

The prevalence of organ specific disease manifestations (nasopharyngitis, conjunctivitis, enteritis) and presumed bacterial complications (pneumonia, otitis media, Group A Streptococcus tonsillitis) was statistically similar (*P* = NS) in influenza positive (*N* = 121) and influenza negative (*N* = 134) patients

### Prevalence of confounding variables in influenza positive patients. Vaccination with Influenza vaccine

Patients presenting with ILI who received the influenza vaccine (39 %) were less likely to test positive for influenza, compared with patients who were not vaccinated (51 %) for Influenza, although the difference was not statistically significant (*P* = 0.064). However, prevalence of viral or bacterial infections (pneumonia, nasopharyngitis, otitis media, Group A Streptococcal tonsillitis, conjunctivitis and enteritis) was similar in both influenza positive and influenza negative patients based on vaccination status (*P* = NS) (Table 3).

**Table 3:** Prevalence of disease manifestations and complications by vaccination status in influenza positive patients

	Pneumonia	Nasopharyngitis	Otitis Media	Group A Streptococcus tonsillitis	Conjunctivitis	Enteritis
Vaccinated	0.421	0.094	0.638	0.532	0.837	0.042
Not Vaccinated	0.953	0.361	0.553	0.540	0.953	0.195

Prevalence of viral or bacterial infections (pneumonia, nasopharyngitis, otitis media, Group A Streptococcus tonsillitis, conjunctivitis, enteritis) was similar in influenza positive patients (*N* = 121) based on vaccination status (*P* = NS)

## Discussion

The current study demonstrates that in a primary care setting: (1) the prevalence of organ specific disease manifestations (conjunctivitis, enteritis) and presumed bacterial complications (pneumonia, otitis media, Group A Streptococcal tonsillitis) was similar in influenza positive and influenza negative patients, (2) patients who were vaccinated with influenza vaccine were less likely to test positive for influenza compared with patients who were not vaccinated, and (3) in patients seeking medical attention for ILI the insurance type or co-payment amount did not significantly affect influenza status. This study suggests that in a primary care setting the prevalence of bacterial/viral complications was similar between influenza positive and negative patients. It is likely that our population represents a milder spectrum of illness; patients that have more severe symptoms or complications due to influenza will seek medical attention in a hospital or emergency rooms.

Laboratory diagnosis of influenza virus can be accomplished by either detection of virus or the patient’s immune response to the virus [8]; diagnosis of influenza is critical for prevention, surveillance, containment, and treatment of the disease [8]. It is difficult to establish a positive influenza diagnosis based on clinical presentation alone due to the fact that other respiratory viruses in both children and adults may cause similar nonspecific symptoms which can co-circulate during influenza outbreaks [8]; diagnosis of influenza based solely on clinical presentation may be problematic [9, 10]. Thus, due to the variability of its presentation, a reliable clinical diagnosis of influenza may be difficult; rapid diagnostic tests are available to assist the medical provider to make a definitive influenza diagnosis [11]. A prompt diagnosis is important; initiation of antiviral therapy (e.g. Oseltamivir) is recommended to prevent infection in at-risk people, and is most effective when administered within the first 48 h of first symptoms of infection [11]. Further, antibacterial therapy may be avoided in the absence of signs and symptoms suggestive of bacterial super infections [11]. Rapid diagnosis of influenza allows for early detection and interventions to be implemented for limiting the scale of possible outbreaks in school and nursing homes [11].

Diagnostic methods for virus identification include detection of influenza viral antigen using immmunoassays (enzyme immunoassay (EIA), immunofluorescence microscopy), point of care (POC) testing (rapid antigen testing (immunochromatographic assays), or optical immunoassays) and detection of viral nucleic acid by use of nucleic acid amplification (polymerase chain reaction) in respiratory tract samples [8]. Alternative laboratory tests include influenza viral isolation or serological detection of influenza antibodies which may take several days or up to a week for test results [11], and therefore may not be useful for acute diagnosis of influenza [11]. Diagnostic kits (EIA/POC testing) can provide results within 1 h of specimen collection [8]. It should be noted that these kits should not be used to predict severity of illness or used for complications of influenza [12]. However, they may be useful as surveillance tools in detecting changes in epidemiology (i.e. outbreaks outside of the typical influenza season, identifying unusual clusters of complications or abnormal events in infectious disease). Recommendations for use of these assays have been issued by the World Health Organization [8, 12].

Compared with other viral respiratory infections (e.g. RSV, Adenovirus, Parainfluenza), influenza may cause a more severe and prolonged illness and is also associated with higher rates of secondary bacterial infections [13]. The most common primary complication of influenza is viral pneumonia [13]; risk factors for development of pneumonia include age (>65 years), lack of previous exposure to influenza virus, history of pulmonary disease, cardiovascular disease, or pregnancy [13]. Prior literature has established that secondary bacterial infections or complications (caused by *Streptococcus pneumoniae, Haemophilus influenza, Staphylococcus aureus, Streptococcus pyogenes*) during previous influenza pandemics contribute to morbidity and mortality [14–16]; the association between primary viral influenza and secondary bacterial pneumonia is well documented [14]. Possible proposed mechanisms include increased colonization of the upper respiratory tract and bacterial-viral synergistic copathogenesis [17, 18]. It has also been suggested that immune-pathogenic responses may be responsible for the synergistic effects of viral and bacterial infection stimulating inflammatory responses [18]. However, it should be mentioned, that receipt of influenza vaccine is associated with a reduced risk of being hospitalized with influenza pneumonia [19].

It is well established that viral co-infections are frequent in children, but the clinical consequences are unclear [20]. Co-infection occurs in children (25-40 %) with bronchiolitis [20, 21]; infection with respiratory syncytial virus (RSV) and metapneumovirus is associated with a 10-fold greater likelihood of PICU level of care [21]. Other studies have reported similar findings with RSV and rhinovirus co-infection [22, 23], while other studies have not confirmed these conclusions [24–26]. The pathogenesis of dual respiratory viral infections is unknown [20]. However, many studies were limited to critical care settings, which may introduce selection bias, due to patient acuity [20].

The next part of our study investigated the prevalence of complications by influenza vaccination status between influenza positive and influenza negative cases. In our cohort, we found that there was a trend for patients who were vaccinated to be less likely to test positive for influenza virus compared with patients who were not vaccinated (*P* = 0.064). While the difference was not statistically significant, this may have been due to small sample size and weak match of vaccine virus to circulating seasonal Influenza Virus. There have been few published studies that have examined the effectiveness of influenza virus vaccine against serious complications in the outpatient setting [27]. However, clinical trials and observational data provide evidence that influenza vaccination is effective in reducing illness due to influenza [27, 28]. Influenza virus vaccines can provide moderate protection against Influenza Virus, but such protection may be reduced or absent in some seasons [28]. The main strategy for control and prevention of pandemic and seasonal influenza has been vaccnation [28, 29]. In addition, vaccination is one of the most cost-effective ways to prevent infection and their complications [30].

The last part of our study evaluated the effect of the type of insurance and co-payment amounts on the likelihood of seeking medical treatment with influenza virus infection. We observed that 53 % of patients who tested influenza positive were required to pay a co-payment ($10-$50) for their doctor’s visit, while 47 % of patients who tested influenza positive were not required to pay a co-payment ($0) for their doctor’s visit (*P* = 0.194). Therefore, we did not find any indication that co-payment or type of insurance influenced the decision of patients to seek medical attention. However, a patient with a high co-payment may initially hesitate to seek medical attention, unless absolutely necessary. Such patients may go directly to the emergency room, and thus, we were unable to measure this variable. It has been reported that implementation of the patient protection and affordable care act was associated with increased health insurance coverage for 19 to 25 year olds without changes in health status or perceived health care affordability or use of flu vaccination [31]. However, insured patients were better off than their uninsured counterparts with respect to access to care, affordability, and health care use [31]; the findings underscore the idea that insurance may be necessary, but not sufficient to alter overall health and use of health care [31]. It should be noted that any plausible effect of health insurance on health status in the general population will most likely be small and easily confounded by selection effects in observational settings [32]. Further research on this topic will require study designs based on larger samples than those which are usually available for health services research [32].

This retrospective study has several limitations, including small sample size, lack of radomization, and lack of formal severity score for each patient’s illness or complication. In addition, a retrospective/observational study may be more prone to selection bias. It could also be, that patients with an illness too mild to cause significant illness may never have visited the doctor’s office and could not be captured due to our retrospective study design. Lastly, this study is a limited-site study and should be confirmed in larger-scale population based studies. However, this study has several strengths, including continuity and follow-up within the same practice, and the fact that data used in this study are less likely to be affected by recall bias or subjective parental interpretation because diagnoses and clinical information were obtained and documented by a physician during clinical encounter and were not parent-based.

## Conclusions

In a primary care setting, complications from viral or bacterial infections do not statistically differ in influenza positive compared with influenza negative patients presenting with ILI. Vaccination with influenza vaccine may decrease duration or severity of symptoms and remains an important public health intervention. In addition, the type of health insurance or co-payment amount did not affect seeking attention for influenza. The use of testing remains a helpful tool for identifying influenza positive patients for the purpose of treatment and prevention efforts.

## Abbreviations

ILI, influenza-like illness; POC, point of care.
